# Transcriptome-Based Traits of Radioresistant Sublines of Non-Small Cell Lung Cancer Cells

**DOI:** 10.3390/ijms24033042

**Published:** 2023-02-03

**Authors:** Margarita Pustovalova, Philipp Malakhov, Anastasia Guryanova, Maxim Sorokin, Maria Suntsova, Anton Buzdin, Andreyan N. Osipov, Sergey Leonov

**Affiliations:** 1 School of Biological and Medical Physics, Moscow Institute of Physics and Technology, 141700 Dolgoprudny, Russia; 2 World-Class Research Center “Digital Biodesign and Personalized Healthcare”, Sechenov First Moscow State Medical University, 119435 Moscow, Russia; 3 Shemyakin-Ovchinnikov Institute of Bioorganic Chemistry, 117997 Moscow, Russia; 4 State Research Center-Burnasyan Federal Medical Biophysical Center of Federal Medical Biological Agency (SRC-FMBC), 123098 Moscow, Russia; 5 N.N. Semenov Research Center of Chemical Physics, Russian Academy of Sciences, 117977 Moscow, Russia; 6 Institute of Cell Biophysics, Russian Academy of Sciences, 142290 Pushchino, Russia

**Keywords:** non-small cell lung cancer, DNA repair, radioresistance, ionizing radiation, transcriptomics, gene expression

## Abstract

Radioresistance is a major obstacle for the successful therapy of many cancers, including non-small cell lung cancer (NSCLC). To elucidate the mechanism of radioresistance of NSCLC cells and to identify key molecules conferring radioresistance, the radioresistant subclones of p53 wild-type A549 and p53-deficient H1299 cell cultures were established. The transcriptional changes between parental and radioresistant NSCLC cells were investigated by RNA-seq. In total, expression levels of 36,596 genes were measured. Changes in the activation of intracellular molecular pathways of cells surviving irradiation relative to parental cells were quantified using the Oncobox bioinformatics platform. Following 30 rounds of 2 Gy irradiation, a total of 322 genes were differentially expressed between p53 wild-type radioresistant A549IR and parental A549 cells. For the p53-deficient (H1299) NSCLC cells, the parental and irradiated populations differed in the expression of 1628 genes and 1616 pathways. The expression of genes associated with radioresistance reflects the complex biological processes involved in clinical cancer cell eradication and might serve as a potential biomarker and therapeutic target for NSCLC treatment.

## 1. Introduction

Lung cancer is the leading cause of cancer-related deaths worldwide [1], with non-small cell lung cancer (NSCLC) accounting for ~85% of all cases [2]. Radiotherapy is a critical choice in the curative management of patients with inoperable NSCLC; however, the prognosis remains poor due to radioresistance of cancer cells present in heterogeneous tumor populations [3]. Radioresistance is linked to both intrinsic (cancer stem cells, mutational status, epithelial–mesenchymal transition (EMT) process) and extrinsic (hypoxia, tumor microenvironment) mechanisms [4] in which cancer cells accumulate genetic changes allowing them to survive harsh treatment conditions and repopulate. In this case, understanding the changes in gene expression profiles and signal molecular pathways of radioresistant cells is important for the selection of new treatment options and therapeutic schemes for NSCLC patients.

With the development of Next-Generation Sequencing, RNA expression profiles can improve our understanding of the molecular mechanisms responsible for cancer radioresistance. In addition, combining expression data of single gene products with a molecular pathway activation level (PAL) can lead to the development of more robust biomarkers, as shown in both experiment [5,6] and theory [7]. Thus, RNA-seq profiles can be used as potent predictors of tumor radioresistance and might serve as potential biomarkers for therapeutic targets. 

We previously generated radioresistant sublines of NSCLC from p53 wild-type A549 and p53-deficient H1299 cells [8,9]. We used Illumina NextSeq 550 to sequence the transcriptomes of parental (A549 and H1299) cells and their radioresistant sublines (A549IR and H1299IR) to expand and improve the data. 

Here, we present the changes in gene expression profiles and molecular pathway activation levels of radioresistant NSCLC sublines in relation to their p53 status.

## 2. Results

### 2.1. Establishment of the Radioresistant NSCLC Cells

**Figure 1:**
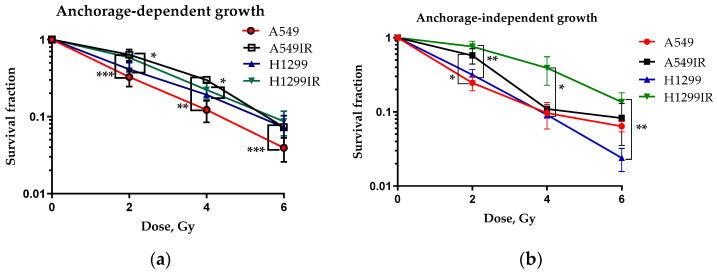
Radiosensitivity of parental and radioresistant NSCLC cells growing in (**a**) anchorage-dependent (solid surface) and in (**b**) anchorage-independent (soft agar) conditions. * *p* < 0.05, ** *p* < 0.01, *** *p* < 0.001. Data are means ± SEM for more than three independent experiments (published previously [10]).

We obtained radioresistant NSCLC sublines using fractionated X-ray irradiation at a total dose of 60 Gy; their survival curves have been previously published [10]. We performed both conventional clonogenic test and the soft agar colony formation assay for assessing cellular radiosensitivity (Figure 1). Both A549IR (wild-type p53) and H1299IR (p53-deficient) cells showed reduced radiosensitivity compared to parental cells growing on a solid surface (Figure 1a). To elucidate further the ability of cells to grow independently of a solid surface, we performed the soft agar colony formation assay (Figure 1b). While A549IR cells showed a statistically significant reduction in radiosensitivity only after exposure to 2 Gy, H1299IR cells showed an overall reduction in radiosensitivity compared to parental H1299 cells (Figure 1b), suggesting their ability to escape from anoikis. Thus, our results demonstrate the overall p53-independent decrease in radiosensitivity of NSCLC cells surviving multifractionated X-ray irradiation. 

### 2.2. Differential Gene Expression of NSCLC Cells 

**Figure 2:**
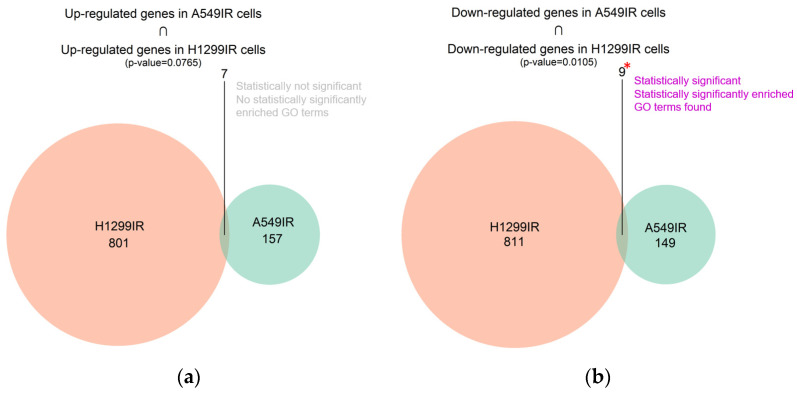
Differential gene intersection showing: (**a**) up-regulated genes in A549IR cells ∩ H1299IR cells; (**b**) down-regulated genes in A549IR cells ∩ H1299IR cells. Red asterisk indicate statistical significance * *p* < 0.05.

To evaluate gene expression changes of radioresistant sublines compared to parental ones, we performed RNA sequencing using the Illumina Nextseq 550 System. The results demonstrated differential expression of 322 genes (log10(control) > 1, |log2FC| > 1) in p53 wild-type radioresistant compared to parental A549 cells. Among them, 164 genes were up-regulated, while 158 genes were down-regulated (Supplementary Table S1). In p53-deficient NSCLC cells, 1628 genes were expressed differentially (log10(control) > 1, |log2FC| > 1) in radioresistant vs. parental cells: 808 genes were up-regulated, while 820 genes were down-regulated (Supplementary Table S1). Radioresistant sublines demonstrated seven common up-regulated genes (*ATRNL1*, *CA2*, *CNR1*, *FAM189A1*, *GFRA1*, *RASGRP1*, *RGL3*); however, no statistically significant enrichment was found (Figure 2a). The nine down-regulated genes that were common between A549IR and H1299IR cells included *ADGRF1*, *EPHA7*, *LOX*, *LY6G5C*, *NSUN7*, *SLC22A31*, *SNAI2*, *TNFRSF11B* and *ZNF233* (*p* = 0.0105) (Figure 2b).

Within the obtained gene sets, Gene Ontology (GO)-based functional analysis provides statistically enriched GO terms that show gene relationships according to three ontology categories and described gene products. These categories are biological process, molecular function and cellular component [11]. We then identified significantly enriched GO terms and characterized radioresistant vs. parental A549 and H1299 cells. In total, enriched GO terms were found in 80 subcategories under biological process, three subcategories under cellular component, and seven subcategories under molecular function. In biological process ontology, the results indicated that desmosome organization, regulation of vitamin D biosynthesis and metabolic process, negative regulation of DNA damage response and signal transduction by p53 class mediator, negative regulation of anoikis, apoptosis, regulation of cell adhesion mediated by integrin and regulation of cell morphogenesis were mainly associated with radioresistant A549IR and H1299IR cells. Significant GO enrichment profiles in the biological process (BP) (only the first 30 out of 80 BPs *p*-value less than 0.01) are summarized in Table 1. Enriched GO molecular function terms in radioresistant A549IR and H1299IR cells were oxidoreductase activity, transmembrane-ephrin receptor activity, chemorepellent activity and E-box binding. Through GO analysis, we found that overrepresented GO terms in our radioresponsive gene sets were closely linked to such cellular components as hippocampal mossy fiber to CA3 synapse, integral and intrinsic components of postsynaptic density membrane. 

### 2.3. Radiation-Induced Transcriptome Alteration in Radioresistant NSCLC Cells through Pathway Activation Level (PAL) Analysis

**Figure 3:**
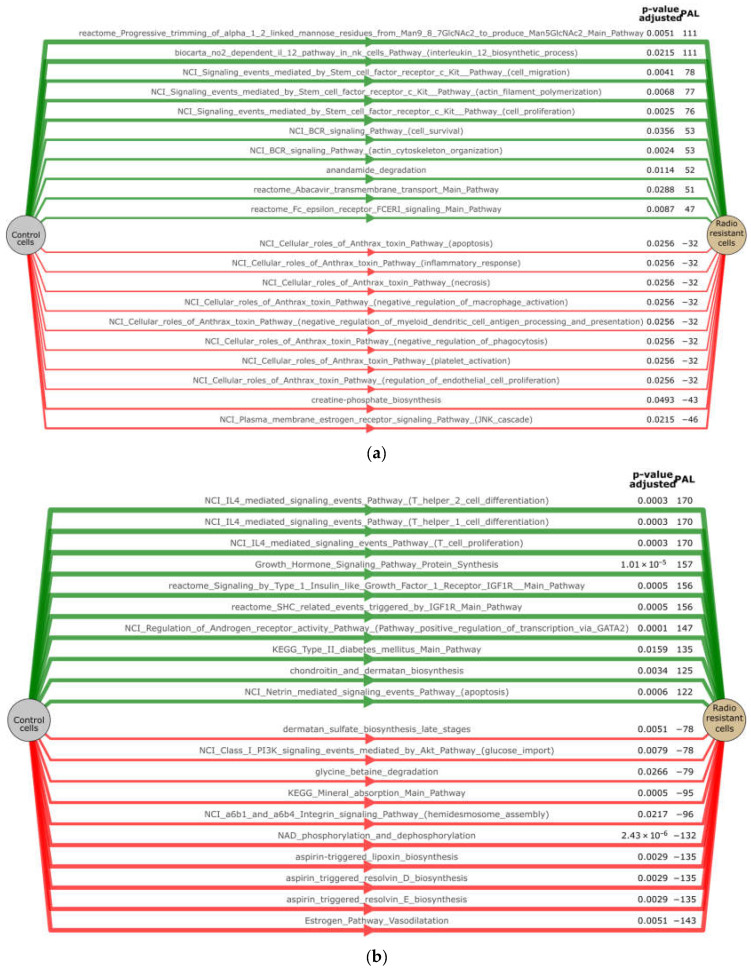
PAL chart of A549 and H1299 cells: (**a**) Top 10 up- and down-regulated pathways in A549IR and (**b**) H1299IR cells, Benjamini Hochberg adjusted *p*-value < 0.05 (only pathways containing 10 and more genes).

Pathway Activation Level (PAL) is an integral parameter, which serves as an accurate qualitative measure of pathway activation [12]. The PAL analysis was performed for functional analysis of our radioresponsive gene sets acquired from the RNA-seq of radioresistant sublines relative to their parental cells using data from Reactome [13], NCI Pathway Interaction [14], Biocarta, KEGG Adjusted, Metabolism and Primary databases. As a result, we found 420 differential pathways (Benjamini Hochberg adjusted *p*-value < 0.05) between radioresistant A549IR and parental A549 cells. A total of 230 pathways were up-regulated in radioresistant A549IR cells (PAL > 0) and 190 pathways were down-regulated in A549IR cells (PAL < 0). Here, we present the top 10 up-regulated and down-regulated pathways associated with the radioresistance-related transcriptome alteration in A549IR sublines (*p*-value less than 0.05) (Figure 3a,b). Based on the results of the PAL analysis, we first identified demannosylation (“Progressive trimming of alpha-1,2-linked mannose residues from Man9/8/7GlcNAc2 to produce Man5GlcNAc2”), interleukin 12 biosynthesis, c-Kit pathways and BCR signaling, proposing metabolical, immunological/inflammatory responses, cell migration and actin filament polymerization as pathways that could be meaningfully associated with radiation tolerance of A549IR cells. Among the top 10 down-regulated pathways with lowest PAL-score, Anthrax toxin pathways involving apoptosis, inflammatory responses, necrosis, negative regulation of macrophage activation and phagocytosis were involved in A549IR radioresistance (Figure 3a). 

We found 1616 differential pathways (Benjamini Hochberg adjusted *p*-value < 0.05) between radioresistant and parental H1299 cells: 917 pathways were up-regulated (PAL > 0), while 699 pathways were down-regulated (PAL < 0) (Figure 3). The most significantly up-regulated pathways (*p*-value less than 0.001) associated with H1299IR radioresistance are shown in Figure 3b. Significant up-regulation of T cell proliferation and differentiation points to a link between immunological responses and radiation tolerance in H1299IR cells. In addition, the high-scoring functions in the pathways were the insulin-like growth factor 1 receptor (IGF1R) and the diabetes mellitus pathway with subsequent inhibition of the PI3K/Akt pathway involved in glucose import (Figure 3b). The latter was among the top 10 scoring down-regulated pathways considerably involved in H1299IR radioreisistance. 

**Figure 4:**
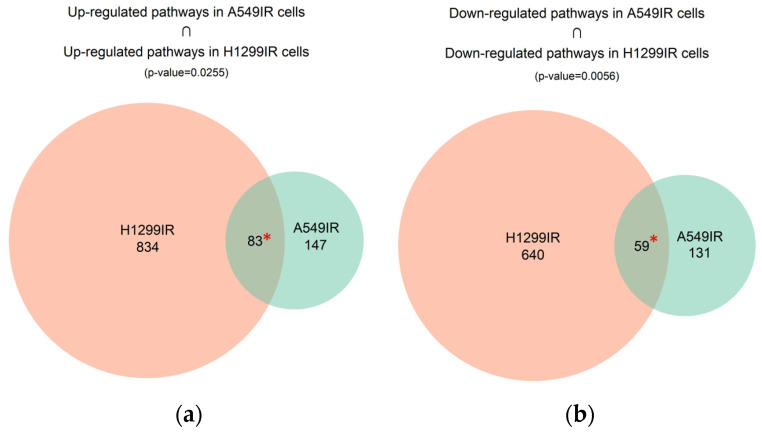
Overlap between differentially regulated molecular pathways between A549IR and H1299IR cells. Overlap of significantly (**a**) up-regulated (PAL > 0) and (**b**) down-regulated (PAL < 0) molecular pathways between A549IR and H1299IR cells are shown; * denotes significance at *p* < 0.05 for the overlaps obtained in perturbation.

The overlap between A549IR and H1299IR up-regulated and down-regulated pathways contained 142 common differential molecular pathways (Supplementary Table S2). Eighty-two pathways were activated and 59 were inhibited. This suggests non-random overlap between the differential pathways in both comparisons, *p* < 0.05 (Figure 4). 

### 2.4. Differential Pathway Changes in NSCLC Cells

**Figure 5:**
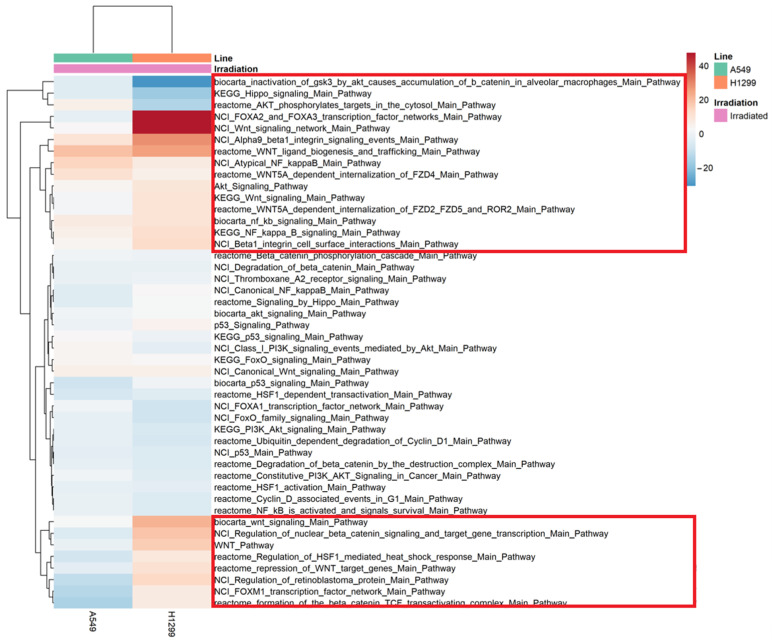
Heat map of RNA-Seq transcriptome analysis for 22 selected pathways in A549IR and H1299IR cells. The most significant differences between H1299IR and A549IR sublines are highlighted in red. Benjamini Hochberg adjusted *p*-value < 0.05.

A main factor related to radioresistance is the presence of cancer stem cells (CSC) inside tumors, which are responsible for metastases, relapses, radiation therapy failure, and a poor prognosis in cancer patients. The Wnt signaling is one of the key cascades regulating maintenance of CSCs, metastasis and immune control of many cancers including NSCLC [15]. We further analyzed the expression of key signaling pathways involved in cancer radioresistance, including Wnt, p53, Hippo, NF-kB, Akt, FOXO, etc. (22 pathways in total) (Figure 5). Heatmap analysis revealed slight up-regulation of beta1 integrin (Alpha9 beta1 integrin signaling events), Wnt (Wnt ligand biogenesis and trafficking, WNT5A-dependent internalization of FZD4) and NF-κB signaling for both A549IR and H1299IR cells (Figure 5, red rectangle). Among pathways of interest, p53, HSF, Cyclin D1, and FOXO almost did not show any change in radioresistant cells over their parental cell lines. 

Interestingly, H1299IR cells demonstrated a significant increase in Wnt signaling over parental level, compared to A549IR subline. “NCI Wnt signaling network Main Pathway” and “NCI FOXA2 and FOXA3 transcription factor networks Main Pathway” were almost 50-fold up-regulated in H1299IR over parental cells, suggesting their role in radioresistance of p53-deficient NSCLC. The level of the main Wnt signaling pathway and Wnt target genes, as well as the “Regulation of HSF1-mediated heat shock response”, “Regulation of retinoblastoma protein” and “FOXM1 transcription factor network”, were significantly up-regulated in H1299IR compared to A549IR cells (Figure 5), suggesting the role of genotype in radioresistance-associated pathway profiles. In contrast, the down-regulation of “Inactivation of Gsk-3 by Akt Causes Accumulation of B-Catenin in Alveolar Macrophages”, “KEGG Hippo signaling pathway” and “Akt phosphorylates targets in the cytosol” were more significantly down-regulated pathways in H1299IR than in A549IR subline.

### 2.5. Charachteristics of Senescence-Associated Radioresistance in NSCLC Cells

It is becoming increasingly clear that acute and chronic senescence are characterized by distinct senescence-associated secretory phenotype (SASP) factors involved in tumor progression. A positive correlation between radioresistance and early induction of head-and-neck squamous cell carcinoma (HNSCC) cell senescence accompanied by NF-κB-dependent production of distinct senescence-associated cytokines was identified by Schoetz et al. [16]. Hence, despite the subtle up-regulation of NF-κB signalling (Figure 5), we attempted to further elucidate the transcription of NF-κB-regulated genes encoding key secretory proteins of SASP. Based on the Chien analysis [17] of 263 genes involved in H-RasV12-induced senescence in IMR-90 normal lung fibroblasts, a well-characterized system of cellular senescence, we analyzed changes in the expression of select NF-κB-regulated genes that encode secretory proteins in our radioresistant sublines. In total, we analyzed the expression of 13 genes (Table 2). Only H1299IR showed significant up-regulation of *CXCL8*, a gene coding IL8, a ligand for the chemokine receptor *CXCR2*, and *TGFB2*, the gene encoding a secreted ligand of the TGF-β (transforming growth factor-beta) superfamily of proteins. Both A549IR and H1299IR sublines demonstrated significant increase in the expression of *IL6*. 

**Figure 6:**
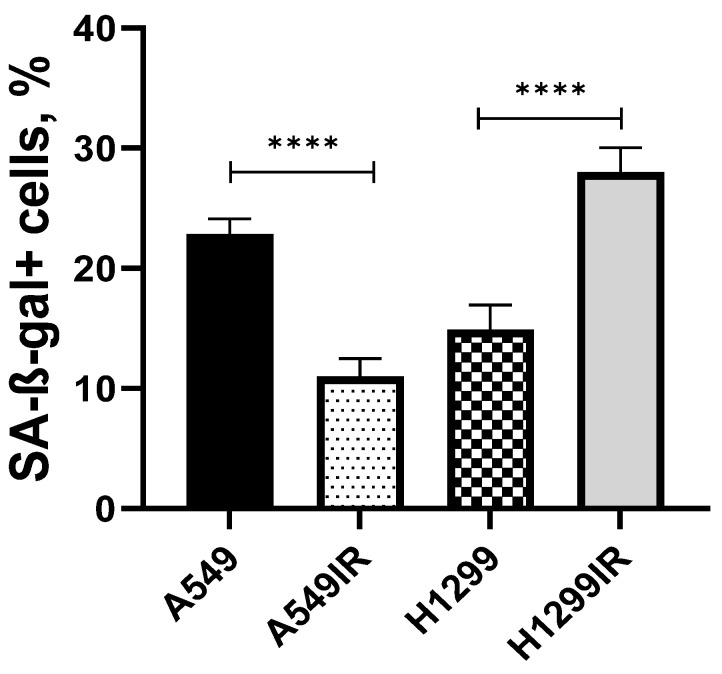
Changes in the proportion of SA-β-Gal positive cells in A549IR and H1299IR. Data are means ± SEM for more than three independent experiments. **** *p* < 0.0001.

However, the expression of *IL6* alone was not accompanied by the increase in the proportion of SA-β-Gal positive cells in A549IR vs. A549 (Figure 6). In contrast, H1299IR cells had a significantly higher (**** *p* < 0.001) fraction of SA-β-Gal positive cells compared to the parental subline. Collectively, our findings emphasize the emerging role of secreted factors (IL8, IL6, TGF-β and Neuregulin 1) in regulating senescence through paracrine and/or autocrine mechanisms conferring radioresistance in NSCLC cell lines.

## 3. Discussion

The present study used whole-genome expression profiles to characterize the radioresistance of A549IR and H1299IR NSCLC cells. The integrative analysis of the expression RNA profiles revealed deregulated biological processes or pathways that may serve as predictors of radiosensitivity and future prospective therapeutic targets. To investigate the gene expression profiles and molecular pathway activation levels, two NSCLC radioresistant sublines (A549IR and H1299IR) with different p53 status were established by fractionated irradiation in a total dose of 60 Gy. We previously reported the activation of pro-survival signaling DNA repair pathways in A549IR and H1299IR cells, including G2/M cell cycle progression, BRCA1 pathway, ATR and DNA double-strand breaks repair by homologous recombination and non-homologous end joining [10]. The key molecular and cellular characteristics of radioresistant cells, including DNA repair, proliferation, epithelial-to-mesenchymal transition, etc., were confirmed and characterized in our previous studies [8,9,18,19].

Recent studies using transcriptomic analysis suggested the role of lncRNAs, such as H19 [20], circRNAs, such as ZNF208 [21], whole-genome miRNA and mRNA [22] in determining NSCLC radioresistance. This demonstrates that transcriptomics is a valuable readout for evaluating outcomes in NSCLC patients undergoing radiotherapy [23]. Here, we interrogated the gene expression profiles obtained by sequencing of total RNA isolated from parental (A549 and H1299) and radioresistant (A549IR and H1299IR) NSCLC cells differing in their p53 status. 

We observed up-regulation of 164 and 808 genes in radioresistant A549IR and H1299IR cells, respectively. Aiming to identify potent common biomarkers of NSCLC radioresistance, we observed seven common up-regulated genes (*ATRNL1*, *CA2*, *CNR1*, *FAM189A1*, *GFRA1*, *RASGRP1*, *RGL3*) and nine common down-regulated genes (*ADGRF1*, *EPHA7*, *LOX*, *LY6G5C*, *NSUN7*, *SLC22A31*, *SNAI2*, *TNFRSF11B* and *ZNF233*) between the two cell lines. Among them, several up-regulated genes are known to be involved in the formation of tumor microenvironments (Carbonic anhydrase 2 (*CA2*)) [24,25], cancer chemoresistance (Cannabinoid receptor 1 (*CNR1*) [26], *GFRA1* [27]) and cancer recurrence after therapy (*RGL3*) [27,28]. Interestingly, some of the common down-regulated genes, such as *EPHA7* and *NSUN7*, are known for both their tumor suppressing and promoting roles, or are associated with overall survival (OS) (*SLC22A31*) [29,30,31,32], while others have clear pro-tumorigenic roles (*ADGRF1*, *LOX*, *LY6G5C*, *SNAI2*, *TNFRSF11B*, *ZNF233*) [30,33,34,35,36]. This may suggest a differential role of many potent pro-tumorigenic genes in cancer aggressiveness and in sustaining radioresistant phenotypes of NSCLC cells during normal culture conditions. However, this notion warrants further investigation and the role of each up-regulated and down-regulated gene in NSCLC radioresistance must be confirmed individually.

High-throughput gene expression data allows calculating pathway activity levels (PAL) associated with cancer radioresistance. In A549IR cells, one of the most activated pathways included progressive trimming of alpha-1,2-linked mannose residues from Man9/8/7GlcNAc2 to produce Man5GlcNAc2 (Figure 4a). N-glycan demannosylation is a highly conserved mechanism allowing cells to avoid protein misfolding and subsequent functional deficiency and cellular toxicity [37]. This process occurs in the Golgi apparatus and forces irreparable misfolded glycoproteins into the Endoplasmic Reticulum-Associated Degradation (ERAD) pathway, where they can be destroyed [38]. Our results demonstrate for the first time the association of the Man-trimming pathway with NSCLC radioresistance.

Surprisingly, we observed the NO2-dependent IL-12 Pathway in NK cells (Figure 4a) as the second most activated pathway in A549IR cells. The IL-12 is a cytokine that activates the large granular lymphocytes or natural killer cells (NK) and is considered a strong candidate for immunotherapy-based tumor cell killing [39]. At the same time, Single Nucleotide Polymorphisms (SNPs) in the IL-12 gene have been associated with the risk of NSCLC [40,41] and breast cancer [42]. 

The overall effect of senescence on cancer progression and cancer cell resistance to X-ray radiation is still not fully understood and remains controversial. Senescence is a state where cells neither function normally nor die. Cells that are damaged or old may enter this suspended state. In this state, they do not reproduce, but are able to communicate with the tumor microenvironment (TME) through senescence-associated secretory phenotype (SASP) in a paracrine fashion [43]. Albeit stress-induced senescence is generally considered to be a tumor-suppressive mechanism [44], long-term treatment-induced senescence of cells may be harmful. Long-term induction of senescence will produce TME that promotes inflammation and immunosuppression [45]. Hence, induction of cancer cell senescence as a recently suggested new therapeutic strategy against cancer [46,47,48,49] should be context-dependent and evaluated carefully based on the cancer stage. Additionally, it seems to be possible to develop new therapeutic strategies to combine priming (immunization) with IR-induced senescent patient-derived cancer cells with IR treatments. In this regard, our study provides new add-in transcriptomic information about activation of SASP-related genes and signaling pathways in response to certain IR stress-inducing insults of different duration.

Interleukins as secretory proteins are involved in SASP-related radioresistance [50]. Previously reported positive correlation between radioresistance of human HNSCC cell senescence accompanied by NF-κB-dependent production of distinct senescence-associated cytokines [16], together with the up-regulation of NF-κB signaling observed in our study, allowed us to assume their potent role in NSCLC radioresistance. The results of the differential gene expression analysis of NF-κB-regulated secretory proteins revealed the association of *CXCL8*, *IL6* and *TGFB2* up-regulation and *NRG1* down-regulation with the increase in the proportion of SA-β-Gal positive cells in H1299IR subline (Table 2). Notably, *CXCR2/IL8* activation is associated with both NSCLC lymph node metastasis and unfavorable prognosis for patients with NSCLC [51]. TGF-β1 causes a switch from cohesive to single cell motility and intravasation that are essential for blood-borne metastasis in breast cancer [52]. It also mediated neutrophil recruitment that drives NOTCH1 signaling-mediated metastasis in colorectal cancer [53]. In H1299IR cells, *NRG1*, Neuregulin 1 coding gene, was significantly down-regulated (Table 2). Importantly, *MUC1* and NRG1 are controlled by the Eukaryotic Translation Initiation Factor 4 Gamma 1 (EIF4G1) for NSCLC survival and tumorigenesis with clinical relevance [54], while *NRG1* is the major tumor suppressor gene postulated to be on 8p: it is in the correct location, is antiproliferative and is silenced in many breast cancers [55]. Last, but not least, the up-regulation of *IL6* alone seemed to be insufficient to induce senescence, as can be seen by the low SA-β-Gal-positive fraction of A549IR cell subline (Figure 6). Our findings suggest that the CXCR2/ligand-axis activation can be pivotal in driving radiotherapy-induced senescence in NSCLC.

Among other pathways involved in cell migration, proliferation and survival, we observed the up-regulation of anandamide degradation pathways in the A549IR subline. Anandamide is an endocannabinoid, part of a family of biologically active lipids that bind to and activate cannabinoid receptors and play a role in biological activities of both the central and periphery nervous systems [56,57]. Anandamide can be transported into the cell where it is metabolized by cyclooxygenase 2 (COX-2) to prostaglandin-ethanolamides (PG-EAs) [58]. The PGs, for example PGE2, are likely to mediate some of the tumour-promoting effects of COX-2, such as immune response, angiogenesis and cell proliferation [59,60]. An IR-induced increase in *COX-2* expression was previously observed in radioresistant A549 cells [61]. Moreover, targeting COX-2 inhibited malignant proliferation of A549 cells in vivo and in vitro [62]. Thus, our results support previously published data on anandamide degradation pathways in radioresistance.

Most of the down-regulated pathways in A549IR cells contained the Anthrax toxin (Figure 4a). Tumor endothelial marker 8 (TEM8), also known as anthrax toxin receptor 1 (ANTXR1), is highly expressed in cancers. Its expression level was associated with tumor size, primary tumor stage and a poor prognosis in patients with lung adenocarcinoma [63]. The ANTXR1 is expressed on metastatic breast cancer stem cells (CSCs) and functions in collagen signaling, as well as Wnt signaling, ZEB1 expression, and CSC self-renewal, invasion, tumorigenicity, and metastasis [64]. The ANTXR1 was identified as a functional biomarker of triple-negative breast CSCs, and pancreatic ductal adenocarcinoma (PDAC) patients stratified based on the ANTXR1 expression level showed increased mortality and enrichment of pathways known to be necessary for CSC biology, including TGF-β, NOTCH, Wnt/β-catenin, and IL-6/JAK/STAT3 signaling and epithelial to mesenchymal transition, suggesting that ANTXR1 may represent a putative CSC marker [65]. However, based on our data, the ANTXR1 pathway does not play a role in NSCLC radioresistance. 

In H1299IR cells, radioresistance was associated with deregulation of metabolism including PI3K/Akt signaling pathway involved in the development of obesity and type 2 diabetes mellitus (DM) [66]. The DM has been shown to be associated with NSCLC progression, suggesting that pre-existing diabetes is an independent prognostic factor for overall survival (OS) among patients with both diabetes and lung squamous cell lung carcinoma who receive standard treatments [67,68,69]. Women with lung cancer and diabetes had significantly increased risk of overall mortality (HR = 1.27, 95% CI: 1.07–1.50) compared to those without diabetes [70]. However, it is still unclear whether the DM is a risk factor for developing NSCLC or vice versa. Here we demonstrate significant up-regulation of Type 2 diabetes mellitus pathways in H1299IR cells through overexpression of IGF-1R and down-regulation of the PI3K/Akt signaling pathway (Figure 3b). The IGF-1R may activate two major signaling pathways, the PI3K/Akt and MAPK pathways [71]. The activated form of Akt, phosphorylated Akt (p-Akt), may inhibit several proapoptotic factors including glycogen synthase kinase-3 beta (GSK3β) [72], which in turn mediates insulin resistance and human type 2 diabetes [73]. Thus, it is tempting to speculate that up-regulation of DM pathways in H1299IR cells serve as a prognostic biomarker of DM in patients with NSCLC, suggesting that pre-existing NSCLC is a potent inducer of DM leading to a worse prognosis. However, this notion warrants further investigation. 

Unlike A549IR, H1299IR cells demonstrated significant up-regulation of the IL-4 signaling pathway (Figure 3b). The IL-4 plays a role in T cell activation, differentiation, proliferation, and survival [74] and, together with its receptor complex (IL4R), has been studied for their role in epithelial cancer progression, including enhanced migration, invasion, survival, and proliferation [75]. Recently, T cell marker genes were proposed as novel prognostic signatures for lung squamous cell carcinoma (LUSC) patients [76]. 

We observed significant 50-fold up-regulation of Wnt signaling in H1299IR cells. Wnt proteins are secreted glyco-lipoproteins that participate in cell fate determination, proliferation and the control of asymmetric cell division during lung development [77]. It has been suggested that Wnt signaling is also involved in the regulation of cancer stem cells [78] and thus, plays an important role in lung carcinogenesis [79]. The major (canonical) Wnt pathway signals through β-catenin [80]. In the absence of Wnt, β-catenin undergoes proteolytic degradation by β-catenin destruction complex consisting of axis inhibition protein (AX-IN), adenomatous polyposis coli (APC), and glycogen synthase kinase 3 beta (GSK3β) phosphorylates β-catenin [78]. In the presence of Wnt, cytoplasmic levels of β-catenin rise and it migrates to the nucleus where it activates the expression of various target genes, including cyclin D1 [81], c-Myc [82] and survivin [83]. 

To elucidate whether Wnt pathway contributes to radioresistance in NSCLC, we further evaluated the Wnt pathway activation. We observed significant up-regulation of canonical (Wnt/β-catenin) pathway in radioresistant H1299IR cells, but not in A549IR cells (Figure 5). Moreover, this process was accompanied by down-regulation of inactivation of GSK3 by Akt, which can cause accumulation of β-catenin in alveolar macrophages pathway. A GSK3β inactivation influences β-catenin, leading to increases in cell motility and migration. The phosphorylation of GSK3β by Akt results in its inactivation. The down-regulation of this process leads to the presence of activated, non-phosphorylated GSK3β, which in turn can phosphorylate β-catenin at Ser33/37. This results in the proteasomal degradation of β-catenin. In colon cancer cells with hyperactivated canonical Wnt signaling, pharmacological inhibition of the PI3K-Akt signaling leads to a nuclear accumulation of β-catenin and FOXO3a, and subsequently increased cell scattering and metastasis [84]. The FOXA2 and FOXA3 transcription factor networks were also up-regulated in H1299IR cells (Figure 5). In A549IR cells, down-regulation of the Wnt signaling pathway might be associated with upregulation of the *MCC* gene which can suppress cell proliferation and the Wnt/β-catenin pathway in colorectal cancer cells (Supplementary Table S1) [85].

The genes and signaling pathways identified in this study provide new important insights into the mechanisms underlying radioresistance in NSCLC cells. The observed whole genome expression profiles can be linked to molecular and cellular characteristics of radioresistant cells, including decreased radiosensitivity, resistance to cell death, enhanced cell proliferation and migration, and raise interest as potential biomarkers and therapeutic targets for NSCLC treatment. 

## 4. Materials and Methods

### 4.1. Cell Cultures and Irradiation

The A549 (p53 wild-type) and H1299 (p53-deficient cells) cell cultures and their isogenic radioresistant sublines A549IR and H1299IR were cultured in RPMI-1640 medium (Gibco, Thermo Fisher Scientific, Waltham, MA, USA) containing 10% fetal bovine serum (Gibco, Thermo Fisher Scientific, Waltham, MA, USA) supplemented with L-glutamine and antibiotics under standard conditions (37 °C, 5% CO_2_). 

The radioresistant sublines were established after 30 fractions of 2 Gy X-ray exposure using 200 kV X-ray RUB RUST-M1 X-irradiator facility (0.85 Gy/min, 2 × 5 mA, 1.5 mm Al filter, JSC “Ruselectronics”, Moscow, Russia). Cells were irradiated five days a week at room temperature. After reaching a total dose of 60 Gy, cells were cultured at 37 °C in a humidified atmosphere with 5% CO_2_ for up to 3 weeks to recover. The radioresistence was previously confirmed using clonogenic assay [19]. 

### 4.2. Clonogenic Test and Soft Agar Colony Formation

The A549, A549IR, H1299, and H1299IR cells were subjected to single X-ray irradiation at doses of 2 Gy, 4 Gy, and 6 Gy, removed from the plastic surface immediately after irradiation, and plated on Petri dishes 60 mm in diameter in the amount of 150, 500, 1000, and 2000 cells/well, respectively. Petri dishes were incubated at 37 °C in a humid atmosphere with 5% CO_2_ for two weeks to form colonies. After that, the cells were fixed with 100% methanol for 15 min at room temperature, followed by Giemsa staining for 15 min. Only colonies containing more than 50 cells were counted. Seeding efficiency (PE) and survival rate (SF) were calculated using the following equations:


PE = number of colonies formed/number of cells seeded × 100%
(1)

SF = number of colonies formed/(number of cells seeded × PE)
(2)

The ancorage-independent soft agar colony formation assay was performed according to the procedure described Borowicz et al. [86]. Exponentially growing A549, A549IR, H1299, and H1299IR cells were X-rayed at doses of 0 Gy, 2 Gy, 4 Gy, and 6 Gy. Collected by trypsin treatment, cells were mixed with 0.6% purified agar. Cell/agar mixtures were added to 6 well plates pre-coated with 1.0% purified agar in complete medium (1.5 mL agar per well) and allowed to solidify for 30 min at room temperature before being placed in a humidified cell culture incubator at 37 °C. Twice a week, 100 µL of culture medium was added to the wells to prevent the agar from desiccation. After colonies formed (~21 days), they were stained with 0.05% crystal violet and counted manually.

### 4.3. Transcriptomic Analysis

The gene expression level was evaluated by RNA-seq analyses of three biological replicates of each cell line. The InnuPREP RNA Mini Kit 2.0 together with innuPREP DNase I Digest (Analytik Jena, Berlin, Germany) were used to isolate total RNA. To measure RNA concentration, we used the Qubit 4 Fluorometer with Qubit RNA Assay kit. The RNA integrity number (RIN) was measured by TapeStation with RNA ScreenTape reagents (Agilent, Santa Clara, CA, USA). For depletion of ribosomal RNA and library construction, the KAPA RNA HyperPrep Kit with the RiboErase (HMR) kit were used. The KAPA UDI Primer Mixes were used for sample barcoding to allow their multiplexing in a single sequencing run. Library concentrations were measured using the Qubit 4 Fluorometer with the Qubit dsDNA HS Assay kit (Life Technologies, Waltham, MA, USA) and TapeStation with High Sensitivity D1000 reagents (Agilent). The RNA sequencing was performed on the Illumina Nextseq 550 System with reagents for single-end sequencing and read length of 75 bp.

### 4.4. Bioinformatics Analysis

FASTQ read files were analyzed using the STAR software [87] in “GeneCounts” mode using transcriptome annotation from Ensembl (GRCh38 genome assembly and GRCh38.89 transcriptome annotation). In total, expression levels of 36,596 genes were measured. The data was normalized using DESeq2 [88].

Changes in the activation of intracellular molecular pathways of IR-survived cells compared to parental cells were quantified using the Oncobox bioinformatics platform [89]. A total of 38 molecular pathways associated with DNA repair were used for analysis [90].

Pathway activation level (PAL) for each molecular pathway was calculated using the formula:

(3) $${\;\mathrm{PAL}}_{p}=\sum_{n}ARR_{np}\times \ln CNR_{n}÷\sum_{n}{\left|ARR_{n}\right|}_{,}$$

where PAL*p* is the level of activation of the molecular pathway *p*; *CNR_n_* (case-to-normal ratio)—the ratio of gene *n* expression level in a tumor sample under study to an average level for the control group; ‘ln’ is the natural logarithm; the discrete *ARR_np_* value (role of activator/repressor) of gene *n* product in the *p* pathway is determined as follows: *ARR_np_* is −1 if gene product *n* inhibits pathway *p*; 1 if *n* activates pathway *p*; 0 if *n* has ambiguous or unclear role in a pathway *p*; 0.5 or −0.5 if *n* is rather an activator of a pathway or its inhibitor, respectively.

### 4.5. Gene Ontology (GO) Analysis

The GO analysis is a commonly applied method for functional studies of large-scale genomic or transcriptomic data [11]. Function enrichGO from the clusterProfiler package was used to identify significantly enriched GO terms among the given list of genes that are differentially expressed in radioresistant and parental cells [PMID: 22455463]. Statistically overrepresented GO categories with *p*-value < 0.05 were considered significant.

### 4.6. Analysis of Senescence-Associated β-Galactosidase (SA-β-Gal) Positive Cells

The “Cellular Senescence Assay” commercial kit (EMD Millipore, Burlington, MA, USA, Catalog Number: KAA002) was used for quantification of senescence-associated β-galactosidase (SA-β-Gal) positive cells. The cells were stained according to the manufacturer’s protocol. The stained cells were visualized using EVOS^®^ FL Auto Imaging System (Fisher Scientific, Pittsburgh, PA, USA) with 20× objective. The proportion of SA-β-Gal positive cells was calculated manually.

### 4.7. Statistics

Statistics were performed using GraphPad Prism 9.0.2.161 software (GraphPad Software, San Diego, CA, USA). Statistical significance was tested using the Student *t*-test. The results are represented as means ± SEM of more than three independent experiments. Significance levels were denoted by asterisks: * *p* < 0.05, ** p < 0.01, *** *p* < 0.001, **** *p* < 0.0001.

## 5. Conclusions

For the first time, we investigated the transcriptome profile of radioresistant NSCLC sublines of A549 and H1299 cells through RNA-seq and bioinformatic functional analysis. Our research with this new methodological approach may provide a useful guideline for the experimental design of gene expression studies and for exploring novel routes to uncover the complete regulatory network involved in radioresistance. In the present study, we used only two radioresistant NSCLC cell sublines derived from parental A549 and H1299 cells. However, it will be worth investigating the differences of the same set of differentially expressed genes in other tumor cell lines and primary cells isolated from lung tumor tissue. Some of the identified novel targets could be potentially interesting in further studies for sensitization of tumor cells to improve radiation effects. Our research could help to interpret the complicated molecular mechanisms leading to radioresistance. Furthermore, it might contribute to the identification of other target genes for predictive biomarkers, improving radiotherapy.

## Figures and Tables

**Table 1:** Differential genes intersection for biological process ontology for common down-regulated genes in A549IR cells ∩ H1299IR cells.

ID	Description	*p*-Value Adjusted	Genes
GO:0002934	desmosome organization	0.019522	*SNAI2*
GO:0030656	regulation of vitamin metabolic process	0.019522	*SNAI2*
GO:0042368	vitamin D biosynthetic process	0.019522	*SNAI2*
GO:0048251	elastic fiber assembly	0.019522	*LOX*
GO:0048755	branching morphogenesis of a nerve	0.019522	*EPHA7*
GO:0070561	vitamin D receptor signaling pathway	0.019522	*SNAI2*
GO:0072178	nephric duct morphogenesis	0.019522	*EPHA7*
GO:1905809	negative regulation of synapse organization	0.019522	*EPHA7*
GO:0061314	Notch signaling involved in heart development	0.019522	*SNAI2*
GO:0033629	negative regulation of cell adhesion mediated by integrin	0.019522	*SNAI2*
GO:0042362	fat-soluble vitamin biosynthetic process	0.019522	*SNAI2*
GO:0072176	nephric duct development	0.019522	*EPHA7*
GO:0018158	protein oxidation	0.019522	*LOX*
GO:0035791	platelet-derived growth factor receptor-beta signaling pathway	0.019522	*LOX*
GO:0010839	negative regulation of keratinocyte proliferation	0.019522	*SNAI2*
GO:0042481	regulation of odontogenesis	0.019522	*TNFRSF11B*
GO:0043518	negative regulation of DNA damage response, signal transduction by p53 class mediator	0.019522	*SNAI2*
GO:0003198	epithelial to mesenchymal transition involved in endocardial cushion formation	0.019522	*SNAI2*
GO:0045779	negative regulation of bone resorption	0.019522	*TNFRSF11B*
GO:0048670	regulation of collateral sprouting	0.019522	*EPHA7*
GO:2000811	negative regulation of anoikis	0.019522	*SNAI2*
GO:0032026	response to magnesium ion	0.019522	*TNFRSF11B*
GO:0046851	negative regulation of bone remodeling	0.019522	*TNFRSF11B*
GO:0060973	cell migration involved in heart development	0.019522	*SNAI2*
GO:0009110	vitamin biosynthetic process	0.019522	*SNAI2*
GO:0031290	retinal ganglion cell axon guidance	0.019522	*EPHA7*
GO:0032793	positive regulation of CREB transcription factor activity	0.019522	*ADGRF1*
GO:0034104	negative regulation of tissue remodeling	0.019522	*TNFRSF11B*
GO:0046716	muscle cell cellular homeostasis	0.019522	*LOX*
GO:0150146	cell junction disassembly	0.019522	*SNAI2*

**Table 2:** Differential gene expression of NF-κB-regulated secretory proteins in A549IR and H1299IR cells (statistically significantly up-regulated genes shown in green, down-regulated genes shown in red).

Genes	A549IR LFC	A549IR *p*-Value	H1299IR LFC	H1299IR *p*-Value
*CCL20*	−1.325	0.246	−0.085	0.791
*CCL3*	0.079	0.810	−0.085	0.791
*CXCL1*	0.043	0.895	0.739	0.134
*CXCL2*	−0.448	0.348	−0.037	0.917
*CXCL3*	−0.247	0.348	0.054	0.924
*CXCL5*	0.513	0.119	−0.085	0.791
*CXCL8*	−0.299	0.424	3.544	0.000
*FGF2*	−0.066	0.760	−0.194	0.560
*IL1A*	0.773	0.354	−0.085	0.791
*IL1B*	0.299	0.582	0.283	0.738
*IL6*	1.494	0.006	1.500	0.004
*NRG1*	−0.322	0.212	−2.212	0.009
*TGFB2*	−0.037	0.863	1.579	0.003

## Data Availability

The materials and data are available from the corresponding authors.

## Supplementary Materials

The following supporting information can be downloaded at: https://www.mdpi.com/article/10.3390/ijms24033042/s1.
